# Hypophysitis in a HIV‐Positive Patient With a Syphilis Latent Infection

**DOI:** 10.1155/crie/3692422

**Published:** 2025-12-12

**Authors:** Fernando Sotelo-Díaz, Astrid Paola Hernández-Valdez, Ismael Quintal-Medina, Jesús Cajigas-Silva, Sarbelio Moreno-Espinosa, Luis Esteban Ramírez-González, José Guillermo Flores-Vázquez, Lesly Portocarrero-Ortíz

**Affiliations:** ^1^ Department of Neurosurgery, Instituto Nacional de Neurología y Neurocirugía Manuel Velasco Suárez, Mexico City, Mexico, innn.salud.gob.mx; ^2^ Department of Neuroendocrinology, Instituto Nacional de Neurología y Neurocirugía Manuel Velasco Suárez, Mexico City, Mexico, innn.salud.gob.mx; ^3^ Department of Infectious Diseases, Médica Sur Hospital, Mexico City, Mexico; ^4^ Department of Infectious Diseases, Instituto Nacional de Neurología y Neurocirugía Manuel Velasco Suárez, Mexico City, Mexico, innn.salud.gob.mx

**Keywords:** coinfection, HIV infections/complications, hypophysitis, hypopituitarism, pituitary gland/pathology, syphilis/complications, *Treponema pallidum*/pathogenicity

## Abstract

**Background:**

Hypophysitis is a rare inflammatory disorder of the pituitary gland that may result from primary autoimmune disease or secondary causes such as infection. In people living with HIV, coinfection with *Treponema pallidum* can precipitate pituitary inflammation, leading to panhypopituitarism, visual field compromise, and neurological symptoms.

**Methods:**

Written informed consent was obtained from the patient, with all personal identifiers removed, in compliance with the CARE guidelines.

**Presentation:**

A 50‐year‐old man with known HIV infection and a history of latent syphilis presented to the emergency department with headache, asthenia, adynamia, and visual decline. Laboratory evaluation demonstrated a positive rapid plasma reagin (RPR) test at 1:64, a CD4 count of 391 cells/µL, a viral load of 40,000 copies/mL, and biochemical evidence of panhypopituitarism. Ophthalmologic assessment revealed bitemporal hemianopia. Brain MRI identified a sellar mass compressing the optic chiasm, consistent with an infiltrative pituitary process. The patient was treated with intravenous penicillin and hormone replacement therapy. A follow‐up MRI showed a reduction in pituitary size, and 1 month later he reported a resolution of neurological symptoms and a marked improvement in visual acuity.

**Conclusions:**

The present description illustrates an uncommon manifestation of syphilis‐related hypophysitis in an HIV‐positive individual, managed successfully with antibiotics and hormonal supplementation. Although rarely reported in literature, syphilitic hypophysitis should be considered in HIV‐infected patients presenting with endocrine deficits and visual field abnormalities. With rising rates of HIV–syphilis coinfection, clinicians may encounter this entity more frequently than previously appreciated.

## 1. Introduction

Hypophysitis, an uncommon inflammatory disorder of the pituitary gland, may arise either as a primary condition, such as idiopathic granulomatous hypophysitis, reported in approximately one case per nine million individuals annually, or as a secondary manifestation of systemic diseases and infections, including HIV and syphilis [1]. In people living with HIV, syphilis tends to follow a more aggressive clinical course and is associated with higher rates of neurological complications, particularly in those with high‐risk sexual practices [2, 3]. Conversely, syphilis itself can enhance HIV transmission and viral replication in plasma and cerebrospinal fluid, predisposing to early ophthalmic and neurological manifestations [4]. Although rare, hypophysitis with panhypopituitarism has been described in patients with HIV and concomitant syphilis but only a handful of such cases have been published to date. Here, we report the case of a 50‐year‐old HIV‐positive Mexican male who developed hypophysitis secondary to syphilis infection.

### 1.1. Clinical Presentation

In 2023, a 50‐year‐old Mexican male with a history of multiple sexual partners was evaluated. He had been diagnosed with HIV infection in 2011 and reported moderate adherence to antiretroviral therapy. At the time of presentation, he was receiving bictegravir/emtricitabine/tenofovir alafenamide. In 2017, he had been treated with penicillin for latent syphilis with ocular involvement, although he could not recall the exact dosage or duration of therapy.

He presented to the emergency department with complaints of asthenia, adynamia, mild headache, and decreased visual acuity. Prior to admission, he had been followed at an outpatient HIV clinic in Mexico City, where a lumbar puncture revealed low cellularity (5 cells/µL), elevated protein concentration (97.8 mg/dL), and normal glucose levels (56 mg/dL). Laboratory tests showed a CD4 count of 391 cells/µL and a viral load of 40,000 copies/mL.

**Figure 1:**
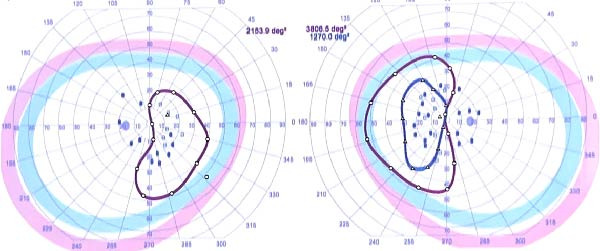
Visual field assessment. Best corrected visual acuity was 20/50 in the left eye and 20/30 in the right eye. Campimetry demonstrated a bitemporal hemianopia, consistent with a chiasmatic syndrome.

On examination, his mental status revealed deficits in short‐term memory and impaired executive function, as he was unable to complete Luria’s tests after three attempts. Neurological examination showed reduced visual acuity (20/30 in the right eye and 20/50 in the left eye) (Figure 1). The Ishihara test was negative, and campimetry demonstrated bilateral heteronymous hemianopia, which was later confirmed by ophthalmology. The remainder of the neurological exam was unremarkable, with no motor, sensory, or meningeal signs.

**Figure 2:**
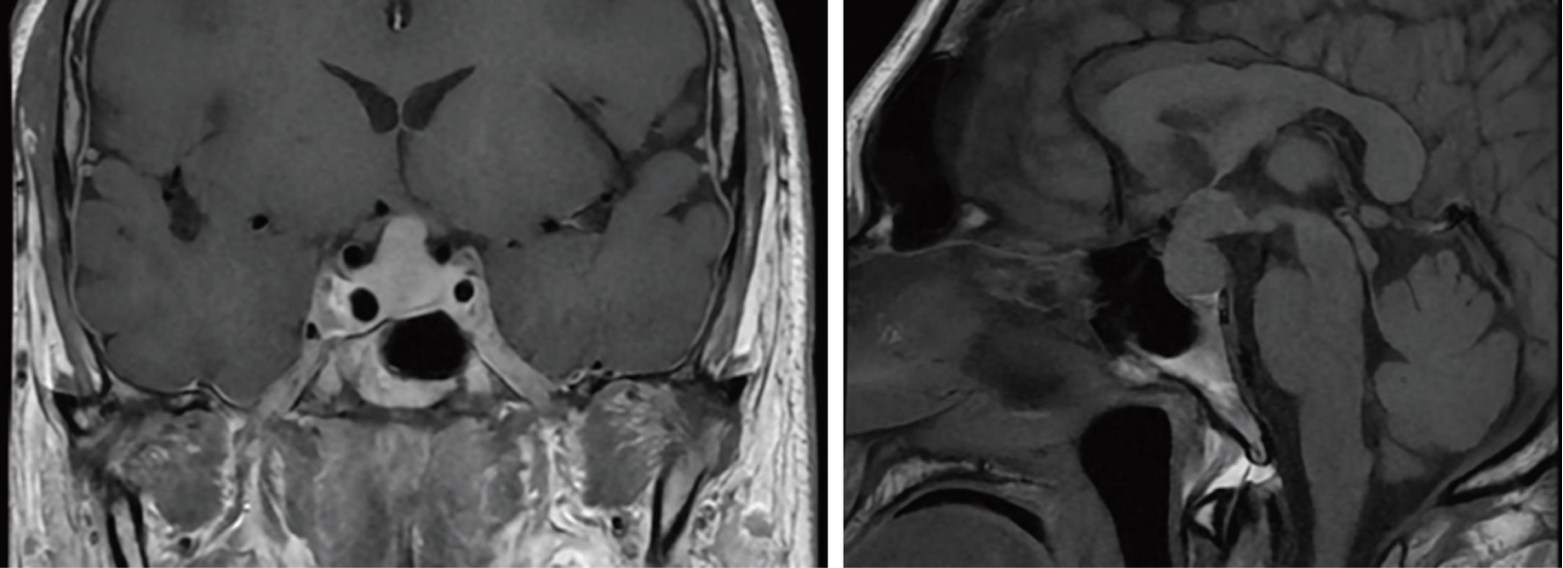
MRI T1WI with gadolinium at admission. Sellar region MRI T1‐weighted image at admission (17.06.2023): MRI T1‐weighted imaging with gadolinium contrast at admission. A bilobed sellar and suprasellar mass with well‐defined margins is observed, extending dorsally and caudally with displacement and compression of the optic chiasm, hypothalamus, and third ventricle. The pituitary gland measured 21 mm × 14 mm × 16 mm, isointense to white matter on T1, and showed avid, homogeneous contrast enhancement.

General laboratory studies were within normal limits. However, given his HIV status and history of syphilis, together with a positive rapid plasma reagin (RPR) test at a titer of 1:64, the Infectious Diseases team initiated intravenous penicillin (4,000,000 units). The patient was admitted to the neurology unit for further evaluation. An MRI with gadolinium contrast performed the following day revealed an infiltrative sellar lesion extending to the optic chiasm, hypothalamus, and third ventricle (Figure 2).

Endocrine evaluation demonstrated panhypopituitarism (Table 1). He was referred to Endocrinology, where oral levothyroxine (100 µg daily) and oral prednisone (10 mg daily) were initiated. During hospitalization, he developed polyuria, polydipsia, and low urine specific gravity consistent with diabetes insipidus secondary to inflammatory pituitary involvement, which was managed with desmopressin.

**Table 1:** Hormonal profile at admission, discharge, and one‐year follow‐up.

Laboratory (normal value)	18.06.2023 (admission)	26.06.2023 (discharge)	12.01.2024 (1 year later)
Luteinizing hormone (2.8–6.8 mUI/mL)	0.28 mUI/mL	0.47 mUI/mL	0.2 mUI/mL
Follicle stimulating hormone (2.8–6.8 mUI/mL)	0.88 mUI/mL	0.66 mUI/mL	0.97 mUI/mL
Prolactin (3.7–17.9 ng/mL)	52 ng/mL	35.0 ng/mL	48.0 ng/mL
Testosterone	0.45 nmol/L	—	0.25 nmol/L
Cortisol	0.99 μg/dL	4.22 μg/dL	5.32 μg/dL
T3 free (4.26–8.1 pmol/L)	2.89 pmol/L	2.87 pmol/L	4.08 pmol/L
T4 free (10–28.2 pmol/L)	6.59 pmol/L	11.5 pmol/L	10.6 pmol/L
Thyroid stimulating hormone (0.465–4.68 uUI/mL)	4.78 uUI/mL	1.55 uUI/mL	2.79 uUI/mL

*Note:* Hormonal profile at admission, discharge, and 1‐year follow‐up. Laboratory values demonstrate panhypopituitarism at admission, with partial recovery at discharge and normalization at 1‐year follow‐up. The patient remained on antiretroviral therapy, oral prednisone (5 mg daily), and levothyroxine (50 µg from Monday to Friday, 100 µg on weekends), with endocrinology follow‐up every 6 months.

**Figure 3:**
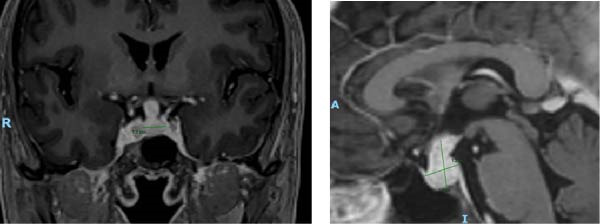
MRI T1WI with gadolinium at discharge. Sellar region MRI T1‐weighted image at discharge (30.06.2023): Findings were consistent with hypophysitis, showing decreased dimensions of the pituitary gland and infundibulum, with reduced compression of the optic chiasm compared with the initial study. The pituitary gland measured 13 mm × 17 mm × 12 mm (rostrocaudal, dorsoventral, and laterolateral axes). Persistent pachymeningeal enhancement was observed, together with mild dural thickening along the clivus and cavernous sinus. No evidence of neuritis was present.

After 15 days of hospitalization and intensive intravenous penicillin therapy, a repeat MRI demonstrated features compatible with hypophysitis, including reduction in pituitary size and infundibular volume, with decreased compression of the optic chiasm compared with the previous image study (Figure 3). Ophthalmological reassessment showed improved visual acuity (20/25 in the right eye and 20/30 in the left eye) and partial recovery of the temporal visual fields bilaterally. Given his clinical and radiological improvement, the patient was discharged with outpatient follow‐up.

At 1‐month follow‐up, he reported improved attention, memory, and visual function, which allowed him to return to work. At 1 year, repeat hormonal evaluation demonstrated normalization of pituitary function (Table 1). He remained clinically stable on antiretroviral therapy, oral prednisone (5 mg daily), and levothyroxine (50 µg from Monday to Friday, 100 µg on weekends).

## 2. Discussion

Syphilitic involvement of the nervous system in individuals living with HIV remains a complex diagnostic and therapeutic scenario. Over the last two decades, the global incidence of *Treponema pallidum* infection has increased, with a particularly marked rise reported in European cohorts [5]. When superimposed on HIV‐related immunosuppression, syphilis tends to progress more rapidly and with greater neurological morbidity. A further challenge lies in the diagnostic yield of serological tests: the Venereal Disease Research Laboratory (VDRL) assay may yield negative cerebrospinal fluid results despite active infection, underscoring the importance of comprehensive clinical and laboratory evaluation in patients presenting with neurological symptoms [1].

Our institution, the Instituto Nacional de Neurología y Neurocirugía in Mexico City, functions as a national referral center where patients with complex neuroinfectious diseases are managed by multidisciplinary teams. This structure facilitated the integrated evaluation of the patient. However, as a publicly funded tertiary care teaching hospital, we also face limitations in access to advanced diagnostic modalities, which represents a constraint in patient work up and management. Notably, our patient developed biochemical and clinical evidence of hypophysitis due to syphilis infection, supported by a positive titer of 1:64 RPR test, a nontreponemal assay detecting antibodies to cardiolipin antigens. Although treponemal tests like TPHA may remain positive for years posttreatment, the high RPR titer and clinical improvement following antibiotic therapy supported the diagnosis of active syphilitic reactivation.

Published reports describing HIV‐associated hypophysitis due to syphilis remain exceedingly rare [6]. Some cases have been attributed to primary pituitary inflammatory processes such as idiopathic granulomatous hypophysitis [4, 7] or necrotizing granulomatous lesions [5], while others occurred in the setting of profound immunosuppression [8, 9]. In this patient, the inflammatory process was presumed lymphoplasmacytic, as suggested by the radiological pattern and clinical response, consistent with secondary hypophysitis of infectious origin. Histopathological confirmation was not obtained, as the diagnosis was established on clinical, serological, and imaging criteria, and the patient exhibited marked improvement without the need for biopsy. To our knowledge, only one prior report has documented hypophysitis secondary to syphilis in an HIV‐negative individual [10]. This scarcity of documented cases provided the rationale for sharing our experience, which we consider a valuable addition to the existing literature.

## 3. Conclusion

This report describes the unusual presentation of hypophysitis in a 50‐year‐old HIV‐positive Mexican man, attributed to concomitant syphilis infection. The diagnosis was supported by a positive RPR test, evidence of panhypopituitarism, and clinical features of chiasmatic involvement. The patient responded favorably to intravenous penicillin combined with hormone replacement therapy, with radiological regression of the pituitary lesion and recovery of visual function, ultimately allowing him to resume his daily activities.

Our experience underscores the need to maintain a high index of suspicion for syphilis‐related hypophysitis in patients with HIV, particularly when visual disturbances or endocrine dysfunction are present. Early recognition and treatment are essential to prevent long‐term neurological and hormonal sequelae.

## Ethics Statement

The authors have nothing to report.

## Consent

Written informed consent was obtained from the patient for publication of this article and accompanying images.

## Disclosure

This case has not been previously presented at any conference or publication. All authors reviewed and approved the final version of the manuscript.

## Conflicts of Interest

The authors declare no conflicts of interest.

## Author Contributions

Fernando Sotelo‐Díaz, Astrid Paola Hernández‐Valdez, Ismael Quintal‐Medina, and Jesús Cajigas‐Silva collected and analyzed the clinical data. Sarbelio Moreno‐Espinosa and Luis Esteban Ramírez‐González contributed to infectious disease evaluation and treatment strategy. José Guillermo Flores‐Vázquez and Lesly Portocarrero‐Ortíz drafted and critically revised the manuscript.

## Funding

No funding was received.

## Data Availability

All data generated or analyzed during this study are included in this article.
